# The Liver X Receptor in Correlation with Other Nuclear Receptors in Spontaneous and Recurrent Abortions

**DOI:** 10.1155/2013/575604

**Published:** 2013-04-15

**Authors:** Julia Knabl, Aurelia Pestka, Rebecca Hüttenbrenner, Torsten Plösch, Regina Ensenauer, Lena Welbergen, Stefan Hutter, Maria Günthner-Biller, Udo Jeschke

**Affiliations:** ^1^Department of Obstetrics and Gynaecology, Campus Innenstadt, Ludwig-Maximilians-Universität München, 80337 Munich, Germany; ^2^Department of Pediatrics, University Medical Center Groningen, University of Groningen, Ctr Liver Digest & Metab Dis, 9700 AB Groningen, The Netherlands; ^3^Research Center, Dr. von Hauner Children's Hospital, Ludwig-Maximilians-Universität München, 80337 Munich, Germany

## Abstract

The liver X receptors (LXRs) have been shown to be crucially involved in maternal-fetal cholesterol transport and placentation. The aim of this study was to investigate the expression pattern and frequency of LXR under normal physiological circumstances and in spontaneous abortion and/or recurrent miscarriage. A total of 29 (12 physiologic pregnancies/10 spontaneous abortions/7 recurrent miscarriages) human pregnancies in first trimester were analysed for LXR expression. Expression changes were evaluated by immunohistochemistry for receptor and quantitative RT-PCR (TaqMan) was performed to determine the level of LXR mRNA expression. We also stained for RXR**α** and PPAR**γ** as possible heterodimers of LXR. LXR expression was downregulated in the syncytiotrophoblast of spontaneous abortion placentas compared to normal pregnancy. In recurrent miscarriage there was a trend for a downregulation. Decidua showed an even stronger downregulation in both groups. In the syncytiotrophoblast we found a positive correlation for the combination of LXR/PPAR**γ** in abortions and a negative correlation for LXR/RXR**α**. In addition, double-immunofluorescence staining showed that LXR as well as RXR**α** and PPAR**γ** are expressed by the extravillous trophoblast. Finally, RXR**α** and LXR showed coexpression in the same extravillous trophoblast cells. To conclude, our data show that LXR expression is decreased in miscarriage.

## 1. Introduction

Multiple regulatory mechanisms (e.g., immunologic, endocrine, and metabolic) are involved in the success of human pregnancy and disturbances in any of these processes can lead to fetal loss. However, 25–50% of reproductive-aged women experience one or more miscarriages. Furthermore, 1–3% of women during childbearing years suffer from recurrent miscarriage (RM), the occurrence of three or more consecutive spontaneous miscarriages regardless of previous live births [1, 2]. In nearly 50% of affected patients, the cause of RM remains unknown and investigations on underlying processes are mandatory [1]. Previous studies of our group showed that nuclear receptors in general are involved in this process. The nuclear retinoid X receptor (RXR)—which is involved in cell proliferation, cell differentiation, and organogenesis [3]—is upregulated in recurrent miscarriages [4]. RXR is a key player of the receptor family, due to its ability to form heterodimers with other nuclear receptors. Heterodimer partners are, for example, peroxisome proliferator-activated receptor (PPAR), thyroid hormone receptor (TR), and liver X receptor (LXR) [5–7]. PPAR*γ* expression and role are already linked to trophoblast invasion [8, 9] and downregulation of RXR*α* is discussed as protection against apoptosis [4]; nothing is known about the role of the nuclear oxysterol receptor—the liver X receptor (LXR)—in miscarriage. 

LXR is a physiological regulator of lipid and cholesterol metabolism that also acts in an anti-inflammatory way. Because LXRs control diverse pathways in development, reproduction, metabolism, and inflammation, they have potential as therapeutic targets [10]. LXRs are expressed in human and mouse trophoblasts and the placenta from early gestation [11] and are regulators of trophoblast invasion [12] and maternal-fetal cholesterol transport [13, 14] which makes them key players for successful placentation and embryonic development.

Overall, there is only limited knowledge about the role of nuclear receptors in miscarriage and, therefore, the aim of our study was to investigate the expression pattern of LXR in trophoblast of normal and disturbed pregnancies and to put the LXR changes in context with expression changes in the other nuclear receptor RXR*α* and PPAR*γ*.

## 2. Materials and Methods

### 2.1. Patient Data

Tissue samples from spontaneous abortions (*n* = 10), recurrent miscarriages (*n* = 7), and legal termination of pregnancy (*n* = 12) at gestational weeks from 7 to 12 were analysed (see Table 1). Samples were obtained by dilatation and evacuation without any prior pharmaceutical induction. In cases of spontaneous abortion, evacuation was performed within the first 24 h after diagnosis. All women included in the study had a null medical and family history. History taking was systematic, aiming to exclude, apart from common disorders, possible implication of clotting disorders and autoimmune diseases, already known as aggravating factors for increased risk for miscarriages. In all samples caryotypic analysis excluded chromosomal abnormalities. Additionally, in all samples microbiology analysis excluded possible intrauterine infection (bacteria, including *Chlamydia trachomatis*). All women had a normal first trimester vaginal swab. 

### 2.2. Immunohistochemistry

Afterwards, the samples were embedded in paraffin wax. Next, the tissue slides were deparaffinised in xylol for 20 min., washed in 100% ethanol, and then incubated in methanol/H_2_O_2_ for 20 min. Rehydration of the slides in an alcohol gradient to distilled water followed. The slides were then placed in a pressure cooker which contained sodium citrate (pH = 6.0). After washing the slides in PBS, they were incubated in power block (BioGenex, Fremont, USA) for 3 min which was diluted 1 : 10 in distilled water. 

Each slide (healthy pregnancy, miscarriage, recurrent miscarriage) was separately incubated with each primary antibody. The primary antibodies which were used for the experiments were anti-LXR rabbit IgG polyclonal antibody, which detects both subtypes of LXR A&B (Lifespan Biosciences, Seattle, USA), anti-human RXR*α* mouse monoclonal IgG2a antibody (1 mg/mL) (clone no. K8508; PPMX, Perseus Proteomics), and anti-PPAR*γ* rabbit polyclonal antibody (Abcam, Cambridge, UK). Anti-LXR (1 mg/mL) was diluted 1 : 200 in power block which was previously diluted 1 : 100 in PBS. Anti-RXR*α* (1 mg/mL) was diluted 1 : 200 in PBS and anti-PPAR*γ* (0.2 mg/mL) was diluted 1 : 1000 in Dako diluting medium. 

Incubation of the sections with the primary antibodies lasted for 16 h at 4°C. After incubation, the sections were washed in PBS twice. This step was followed by incubation of the slides with the secondary antibody for 30 min. For slides which were either incubated with anti-LXR or anti-PPAR*γ*, the secondary antibody from the Vectastain Elite Rabbit IgG Kit (Vector Laboratories, Burlingame, USA) was used. Slides which were previously incubated with anti-RXR*α* were incubated with the Vectastain Elite Mouse IgG Kit (Vector Laboratories). Next, the slides were washed in PBS and then incubated with the ABC complex (Vector Laboratories) for 30 min. Staining of the slides with 3,3-diaminobenzidine substrate solution (DAB) (Dako, Glostrup, Denmark), for 60 sec. in case of anti-LXR detection, 1 min in case of anti-RXR*α* detection, respectively, and 2 min. in case of anti-PPAR*γ* detection followed. Counterstaining of the slides was carried out with hemalaun for 2 min. In between the staining and counterstaining the slides were washed in distilled water for 2 min. twice. Finally, sections were washed in tap water for 5 min. and afterwards dehydrated in an ascending alcohol series and then washed in xylol. Then, the slides were coverslipped with Eukittquick-hardening mounting medium (Sigma Aldrich, Saint Louis, USA). 

The sections were examined by two independent observers using a Leitz Diaplan microscope (Leitz, Wetzlar, Germany). Per slide, ten fields were examined with the semiquantitative immunoreactive score (IRS). The IRS score examines the intensity and distribution of antigen expression and is calculated by multiplying the percentage of positively stained cells (0: no staining; 1 < 10% of the cells; 2: 11–50%; 3: 51–80%, 4 > 81%) with the cells' intensity of staining (0: none; 1: weak; 2: moderate; 3: strong). 

Positive controls were carried out with breast cancer tissue for PPAR*γ* and RXR*α* detection and colon tissue for detection of LXR. Negative controls were performed by replacement of the primary antibodies by species specific isotype control antibodies (Dako).

### 2.3. Real-Time Reverse Transcriptase-PCR (TaqMan-PCR)

#### 2.3.1. RNA Extraction

The NucleoSpin RNAII Kit (Macherey-Nagel, Düren, Germany) was used for the investigation according to the protocol. The RNA from the villous trophoblast tissue of placentas (20 patients) was extracted. In order to quantify the purified RNA a NanoPhotometer (Implen, Munich, Germany) was used. 

#### 2.3.2. Reverse Transcription

The High-Capacity cDNA Reverse Transcription Kit (applied Biosystems, Foster City, CA, USA) was used for reverse transcription which was done in a mastercycler gradient (Eppendorf, Hamburg, Germany). The temperature conditions of the mastercycler were for 10 min. at 25°C, 2 h at 37°C, 5 sec. at 85°C, and 4°C on hold. 

#### 2.3.3. Real-Time RT-PCR

Real-time RT-PCR reactions were performed in quadruplicate in optical 96-well reaction microtiter plates covered with optical caps, in a volume of 20 *μ*L containing 1 *μ*L TaqMan Gene Expression Assay 20x (Hs00172885_m1 for LXRA Exon boundary 6-7 and Hs01027208_m1 Exon boundary 2-3 for LXRB mRNA detection, all Applied Biosystems, Weiterstadt, Germany), 10 *μ*L TaqMan Fast Universal PCR Master Mix 2x (Applied Biosystems, Weiterstadt, Germany), 1 *μ*L (300–900 ng/*μ*L) template and 8 *μ*L H_2_O (DEPC-treated DI water, Sigma, Taufkirchen, Germany). Thermical cycling conditions were 20 sec. at 95°C, followed by 40 cycles of amplification with 3 sec. at 95°C. and 30 sec at 60°C. The ABI PRISM 7500 Fast (Applied Biosystems, Weiterstadt, Germany) was used to perform the PCR assays. 

Quantification was carried out by the ΔΔCt-method using glyceraldehyde phosphate dehydrogenase (GAPDH) or beta-2-microglobulin as housekeeping genes (Hs99999905_m1 assay for GAPDH mRNA detection and Hs00984230_m1 for beta-2-microglobulin mRNA detection, both Applied Biosystems, Weiterstadt, Germany).

### 2.4. Double-Immunofluorescence Staining

Double-immunofluorescence staining was performed in order to localise the nuclear receptors LXR, RXR*α*, and PPAR*γ* and it was furthermore used to analyse the expression of LXR and RXR on the same placental tissue. Double-immunofluorescence staining was carried out on placentas of healthy pregnancies and spontaneous miscarriages, both groups from the first trimester. For 20 min. the sections were deparaffinised in xylol and after washing them in ethanol they were incubated in ethanol/methanol again for 20 min. Next, the slides were rehydrated in an alcohol gradient and then placed in a pressure cooker with sodium citrate (pH = 6.0). Washing of the slides in PBS followed and then the slides were blocked with ultra V blocking solution (Labvision) for 15 min. The slides were either incubated with anti-LXR*α*/*β* rabbit IgG, diluted 1 : 200, polyclonal goat anti-RXR*α* IgG (AbD Serotec, Oxford, England), diluted 1 : 1000, or polyclonal rabbit anti-PPAR*γ* antibody (Abcam), diluted 1 : 1000. Each section was additionally incubated with HLAG mouse IgG1 (clone MEM-6/9) (AbD Serotec) which was diluted 1 : 50. For all slides incubation took place for 1 h. Sections were then incubated with the secondary antibodies. For LXR and PPAR*γ* the slides were incubated with the Cy3-labelled goat anti-rabbit IgG antibody (Dianova), which was diluted 1 : 500, and the Cy2-labelled goat anti-mouse IgG antibody, diluted 1 : 100. For RXR*α* the Cy3labelled donkey anti-goat IgG antibody (Dianova), which was diluted 1 : 500, and the Cy-2-labelled rabbit anti-mouse IgG antibody, diluted 1 : 100, were used. Next, the slides were embedded in DAPI-containing mounting buffer (Vector Laboratories). Afterwards, the slides were analysed with a fluorescent Axioskop photomicroscope (Zeiss, Oberkochen, Germany). Pictures were taken with a digital Axiocam camera system (Zeiss). 

For double-immunofluorescence staining of LXR and RXR*α* on the same placental tissue the sections were processed like the previous immunohistochemical slides. The primary antibodies which were used included the anti-LXR*α*/*β* rabbit IgG (Lifespan Biosciences), diluted 1 : 200, and the polyclonal goat anti-RXR*α* IgG (AbD Serotec), diluted 1 : 50. Next, the secondary antibodies, the Cy3-labelled goat anti-rabbit IgG (Dianova), diluted 1 : 500, and the Cy-2-labelled goat anti-mouse IgG antibody, diluted 1 : 100, were applied in the slides. The following steps were carried out as described above. 

### 2.5. Statistics

The SPSS/PC software package, version 20 IBM Armont USA was used for data collection and processing as well as analysis of statistical data. It was performed with the nonparametric Spearman's rank correlation coefficient which analyses the statistical dependence between two monotonic, nonlinear variables. Values with *P* < 0.05 were considered statistically significant. Additionally, the Kruskal-Wallis test was used to compare more than two independent groups and in addition the Mann-Whitney-*U* test was used for evaluation of two independent groups. These tests are one-way analysis of variance and analyse two or more samples which are independent from each other. 

## 3. Results

### 3.1. Evaluation of LXR Staining in Abortive Placental Tissue and Controls

**Figure 1:**
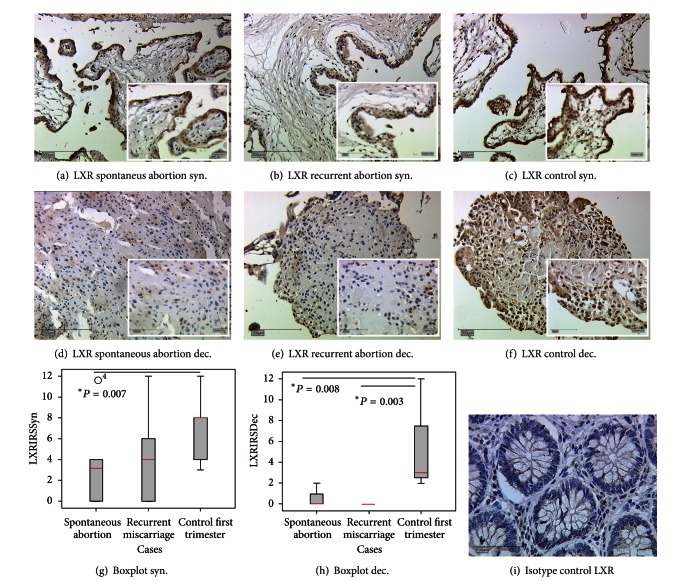
Sections of LXR expression in the syncytiotrophoblast and decidua of placentas from spontaneous abortion ((a), (d)), recurrent miscarriage ((b), (e)), and in regular pregnancies ((c), (f)). (i) shows Isotype control for LXR from colon tissue. Box plots represent the LXR expression in the placental syncytiotrophoblast (g) and decidua (h) of spontaneous abortion, recurrent miscarriages, and control placentas derived immunohistochemically. The boxes display the range between the 25th and 75th percentile and the red horizontal line indicates the median. The bars represent the 5th and 95th percentiles. Circles indicate values which are more than 1.5 times the box length. LXR expression in the syncytiotrophoblast of spontaneous abortion is significantly reduced in comparison to LXR expression in the syncytiotrophoblast of control placentas (_ _*P = 0.007). In the syncytiotrophoblast of recurrent miscarriages a downregulation of LXR expression could be detected, however this trend was not significant (*P* = 0.11). In decidua, we identified significant downregulation of LXR in both spontaneous abortion and recurrent miscarriages with *P* values of 0.003 and 0.008, respectively. The Mann-Whitney-*U* test was used for evaluation of two independent groups.

We identified the expression of LXR in nuclei of cells in the decidua and the syncytiotrophoblast both in regular pregnancy and miscarriage (Figure 1). LXR expression was significantly reduced in the syncytiotrophoblast of spontaneous abortion compared to normal pregnancy (IRS 8 versus 3, *P* = 0.007) (Figure 1(a)). In recurrent miscarriage, we identified the same trend although not significant (IRS 8 versus 4, *P* = 0.11) (Figure 1(a)). In decidua, we found an even stronger downregulation in both groups (recurrent miscarriage (IRS 3 versus 0; *P* = 0.003) and spontaneous abortion (IRS 3 versus 0; *P* = 0.008) (Figure 1(b)).

### 3.2. Real-Time RT-PCR (TaqMan)

**Figure 2:**
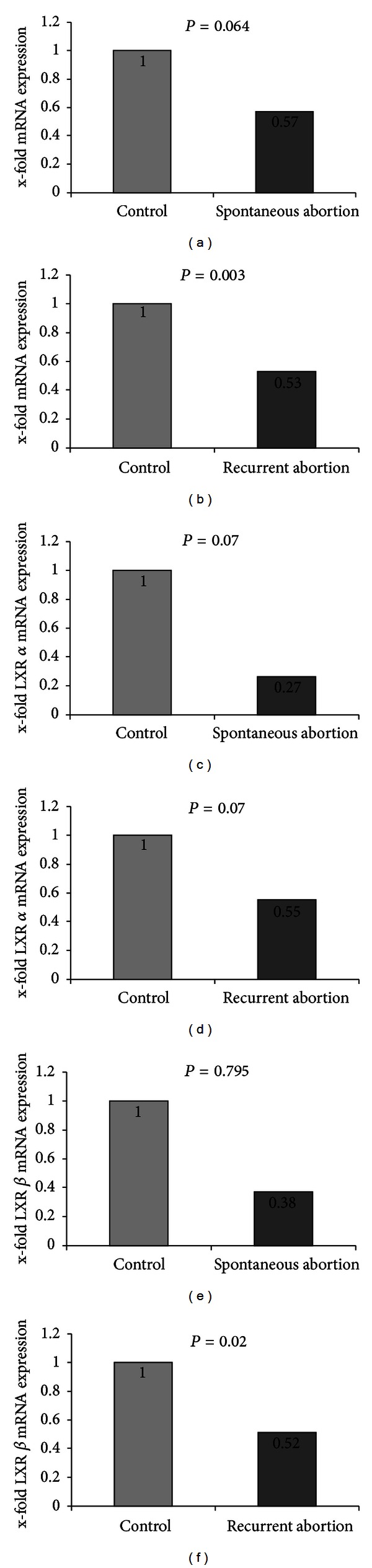
Quantification of mRNA expression of LXR A and B in placentas of spontaneous miscarriages and recurrent miscarriage. (a) We identified a nonsignificant downregulation of LXR mRNA expression in spontaneous abortion placentas compared to normal controls (57%, *P* = 0.064). (b) In recurrent abortion we identified a downregulation of LXR mRNA expression to 53% compared to control placentas. (c) LXR A was downregulated to 27% (*P* = 0.07) and (e) LXR B was downregulated to 38% (*P* = 0.795). (d) LXR A was downregulated to 55% (*P* = 0.07) and (f) LXR B was downregulated to 52% (*P* = 0.02). Mann-Whitney-*U* test was used for evaluation of two independent groups.

Results of quantitative real time RT-PCR (TaqMan) showed that LXR mRNA expression was reduced in placentas of spontaneous miscariages in comparison to healthy villous tissue (Figure 2(a)) however the reduction was not significant (*P* = 0.064). In recurrent miscarriages, a significant reduction in LXR mRNA expression compared to control placentas could be shown (*P* = 0.003) (Figure 2(b)). For quantification of LXR mRNA expression, mean values for LXRA and LXRB were used.

### 3.3. Evaluation of LXR, RXR*α*, and PPAR*γ* in Serial Section of Decidual Tissue in Abortive Placentas and Controls

**Figure 3:**
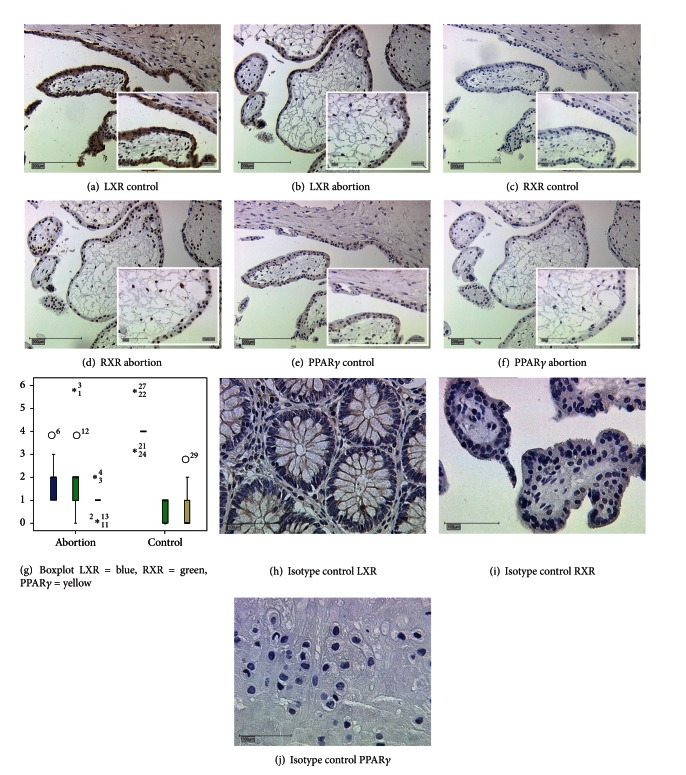
Serial sections of LXR ((a), (b)), RXR*α* ((c), (d)), and PPAR*γ* ((e), (f)) expression in the syncytiotrophoblast of placentas from regular pregnancies ((a), (c), (e)) and in placenta of miscarriage ((b), (d), (f)). All magnifications are 20x lens. Box plots represent the LXR, RXR*α* and PPAR*γ* expression in the placental syncytiotrophoblast of spontaneous abortion and control placentas derived from serial sections (g). (h), (i), (j) show isotype control for LXR, RXR*α*, and PPAR*γ*. The boxes display the range between the 25th and 75th percentile and the horizontal line indicates the median. The bars represent the 5th and 95th percentiles. Circles indicate values which are more than 1.5 times the box length. Asterisks indicate values which are more than 2 times the box length.

**Figure 4:**
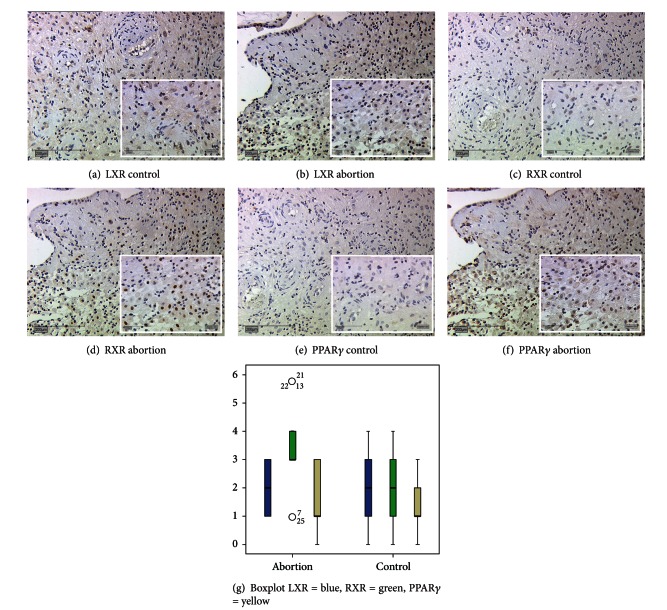
Serial sections of LXR ((a), (b)), RXR*α* ((c), (d)), and PPAR*γ* ((e), (f)) expression in decidua of placentas from regular pregnancies ((a), (c), (e)) and in placenta of miscarriage ((b), (d), (f)). All magnifications are 20x lens. Box plots represent the LXR, RXR, and PPAR expression in decidua of spontaneous abortion and control decidua derived from serial sections (g). The boxes display the range between the 25th and 75th percentile and the horizontal line indicates the median. The bars represent the 5th and 95th percentiles. Circles indicate values which are more than 1.5 times the box length. Asterisks indicate values which are more than 2 times the box length. Isotypic controls for the antibodies are shown in Figure 3.

Serial sections were used to identify synchronized expression of LXR, RXR*α*, and PPAR*γ*. We identified the expression of LXR, RXR*α*, and PPAR*γ* in the nuclei of the syncytiotrophoblast (Figure 3) and decidual cells (Figure 4) of spontaneous miscarriages ((b), (d), (f)) and of first trimester control placentas ((a), (c), (e), all gestational week 7–12). In serial sections besides of reduced expression for LXR in abortive tissue, a significant increase in RXR*α* expression could be observed for the syncytiotrophoblast of miscarriages (IRS 2 for miscarriages, IRS 1 for control placentas, *P* = 0.003). In decidual cells of early pregnancy miscarriages the RXR*α* expression was also increased in comparison to control placentas (IRS 3 for miscarriages versus IRS 2 for control placentas, *P* = 0.006). PPAR*γ* expression in the syncytiotrophoblast of miscarriages was increased in comparison to the control. 

### 3.4. Double-Immuneofluorescence Staining

#### 3.4.1. Identification of Cells in the Decidua Expressing LXR, RXR*α*, and PPAR*γ*

**Figure 5:**
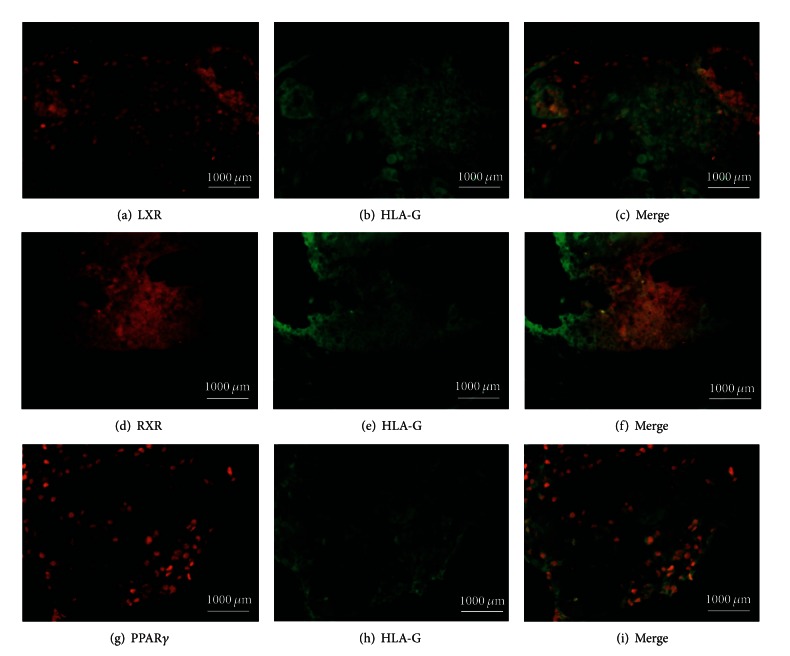
Identification of cells in the abortive decidua expressing nuclear receptors. Expression of LXR (a), RXR*α* (d), and, PPAR*γ* (g) is shown in red. Expression of HLA-G as a marker for extravillous trophoblast cells is shown in green ((b), (e), (h), resp.). Coexpression of both, nuclear receptor in red and HLA-G in green, indicates extravillous trophoblast cells expressing LXR (c), RXR*α* (f), and PPAR*γ* (i). The same expression scheme was identified in normal control decidua.

HLA-G was used as antigen that is expressed exclusively in extavillous trophoblast cells. Expression of LXR, RXR*α*, and PPAR*γ* is shown in Figures 5(a), 5(d), and 5(g), respectively, in abortive tissue. The pictures showing expression of HLA-G are shown in Figures 5(b), 5(e) and 5(h), respectively. Double-immunofluorescence staining as shown in Figures 5(c), 5(f), and 5(i), respectively, indicates synchronous expression of both nuclear receptor and HLA-G in the placentas of miscarriages. We identified the same expression scheme in normal control decidua.

#### 3.4.2. Identification of Cells in the Decidua Expressing LXR and RXR*α* Together

**Figure 6:**
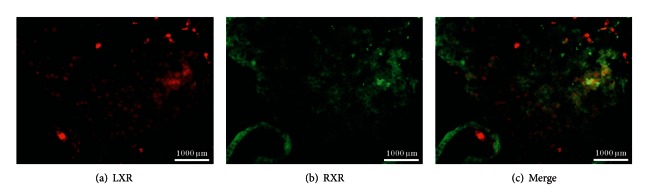
Analysis of LXR and RXR*α* coexpression in extravillous trophoblast cells (EVT) of abortive tissue. LXR expression is shown in red (a). RXR*α* expression is visualized in green (b). Synchronized expression of both nuclear receptors in EVT is shown in (c). The same expression scheme was identified in normal control decidua.

In this study, EVT in placentas from miscarriages expressed LXR, stained in red (Figure 6(a)), and they expressed RXR*α*, stained in green (Figure 6(b)). Triple filter excitation showed expression of LXR and RXR*α* in the same EVT, indicated by yellow staining. Hence, a coexpression of LXR and RXR*α* could be shown in placentas of spontaneous miscarriages (Figure 6(c)). The same applied for the normal control decidua.

#### 3.4.3. Correlation Analysis

It is known that PPAR*γ*, RXR*α*, and LXR are able to form heterodimers; therefore we were interested in coexpression and correlation of these nuclear receptors. So we performed correlation analysis of the IRS Score of the nuclear receptors in a combined form for villous and extravillous tissue.

We correlated the IRS score of the nuclear receptor LXR, PPAR*γ*, and RXR*α* in all miscarriage patients and all control trophoblast. We found significant positive correlation in nuclear receptors in patients with abortions, for the combination of LXR/PPAR (Spearman correlation coefficient *r* = 0.514, *P* = 0.01) an negative correlation for LXR/PPAR (Spearman correlation coefficient *r* = −0.51, *P* = 0.01).

## 4. Discussion

Although early pregnancy loss is a common complication of human reproduction, a significant proportion of miscarriages still happen for unknown reasons [2].

To our knowledge, this is the first evaluation of the nuclear receptor LXR and its putative role in spontaneous abortion and recurrent miscarriages. 

We found a strong downregulation of LXR in decidua of both spontaneous and recurrent miscarriage; literally in recurrent miscarriages we found no expression at all. Additionally, we were able to confirm these findings on mRNA level. 

LXR immunostaining was reduced in both spontaneous and recurrent abortions. However, we found a significant reduction in the syncytiotrophoblast of spontaneous abortions, but a trend in the syncytiotrophoblast of recurrent abortions. 

In the first trimester, the maternal blood flow into the intervillous space is restricted creating a low oxygen environment for the trophoblast [15]. A stable O_2_ gradient between maternal decidua and the feto-placental interface is essential in normal villous development, as especially the syncytiotrophoblast is particularly vulnerable to oxidative stress. This is due to the location on the villous surface and the lower concentrations of antioxidant enzymes in early gestation [16, 17]. Defective placentation leads to a premature onset of the maternal circulation and the excessive entry of maternal blood into the intervillous space [18–20]. 

Therefore, downregulation of LXR could be a signal of excessive oxidative stress in the syncytiotrophoblast of spontaneous abortions. In recurrent miscarriage, however, there is a strong immune modulation component, where we speculate that additional mechanisms together with oxidative stress cause abortion [21]. In summary, we identified a strong downregulation of LXR in the extravillous trophoblast and no significant changed expression in the syncytiotrophoblast. Therefore we speculate that pregnancy loss happens in recurrent miscarriage before oxidative damage reached the syncytiotrophoblast layer of the placenta.

Serial section staining as well as double immunofluorescence was used to analyse LXR/RXR*α* and PPAR*γ* co-expression in placental tissue. In addition, HLA-G as marker for the extravillous trophoblast (EVT) [22] was used for identification of LXR/RXR*α*- and PPAR*γ*-expressing cells in the decidua. For nuclear receptor, RXR*α* and PPAR*γ* we also found an upregulation in spontaneous miscarriage. These findings have been already demonstrated in previous studies [4, 8, 23]. 

In addition to the localisation of the nuclear receptors LXR/RXR*α*/PPAR*γ* in villous trophoblasts of these pregnancies, we also found that LXR and RXR*α* showed relevant changes in extravillous trophoblast. We identified LXR and RXR*α* colocalized in extravillous trophoblasts. Furthermore, we found negative correlation for especially LXR/RXR*α* in the syncytiotrophoblast.

LXR activation with synthetic or natural ligands inhibits trophoblast invasion in vitro [24]. Possibly correlation of LXR and RXR*α* might be a sign of increased maternal-fetal cholesterol transport. Plösch et al. showed that LXR upregulation leads to increased expression of the LXR target genes ABCG1 and ABCA 1. This mechanism is considered to increase cholesterol flux from mother to fetus [14]. This might be indicative of pronounced demand in embryogenesis, as cholesterol is crucially involved in neural pattern formation via hedgehog proteins and in brain development [25–27].

To conclude, our data show that LXR expression is decreased in miscarriage and this is attended by changes in correlation changes of LXR with its heterodimer partners RXR*α* and PPAR*γ*, possibly as a result of oxidative stress or proinflammatory processes. 

## Figures and Tables

**Table 1:** Clinical characteristics of the study population.

	Normal pregnancy *n* = 12	Spontaneous abortion *n* = 10	Recurrent miscarriage *n* = 7	*P* value (Kruskal-Wallis test)
Maternal age	33.0 ± 6.7 years (22–41)	31.5 ± 8.8 years (19–43)	34.3 ± 4.6 years (25–39)	0.81
Gestational age	9.0 ± 2.0 weeks (7–12)	9.84 ± 1.4 weeks (7–12)	8.7 ± 2.2 weeks (7–12)	0.37
Gravidity	3.1 ± 2.0 (1–7)	2.2 ± 2.6 (1–9)	2.9 ± 0.8 (2–4)	0.07
Parity	1.2 ± 1.2 (0–4)	1.2 ± 2.6 (0–8)	0.7 ± 10.8 (0–2)	0.47
